# Activation of hypermethylated P2RY1 mitigates gastric cancer by promoting apoptosis and inhibiting proliferation

**DOI:** 10.1515/biol-2022-0078

**Published:** 2023-03-03

**Authors:** Yinggang Hua, Yanling Liu, Long Li, Guoyan Liu

**Affiliations:** Department of Gastrointestinal Surgery, Zhongshan Hospital Xiamen University, Xiamen, China; School of Pharmaceutical Sciences, Xiamen University, Xiamen, Fujian, China; Department of Basic Medicine, Medical College of Xiamen University, Xiamen, Fujian, China

**Keywords:** diffuse gastric cancer, DNA methylation 450K array, P2RY1 receptor, ERK signal pathway, tumor suppressor gene

## Abstract

The P2RY1 receptor is known to cause cancer by activating the ERK signal pathway, and its DNA methylation status and corresponding regulatory mechanism remain unknown. This study used the DNA methylation chip to profile the genome-wide DNA methylation level in gastric cancer tissues. The proliferation and apoptosis of the SGC7901 gastric cancer cell line were determined after treatment with a selective P2RY1 receptor agonist, MRS2365. The promoter region of P2RY1 was found to be highly methylated with four hypermethylated sites (|Δ*β* value| > 0.2) in diffuse gastric cancer and was validated by bioinformatics analysis in the TCGA database. Also, immunohistochemical staining data obtained from the HPA database demonstrated the downregulated expression of proteins encoded by P2RY1 in stomach cancer tissue. The analysis of MRS2365-treated cells by annexin V/propidium iodide staining and caspase-3 activity assays indicated the induction of apoptosis in SGC7901 cells. The P2RY1 receptor activation in human SGC7901 gastric cancer cells via the MRS2365 agonist induced apoptosis and reduced cell growth. High DNA methylation in the promoter region of P2RY1 might have contributed to the reduced expression of P2RY1’s mRNA, which was likely responsible for the “aggressive” nature of the diffuse gastric cancer.

## 1 Introduction

Gastric cancer is a growing concern due to its high prevalence worldwide and the increasing number of deaths related to the disease [1]. While the etiology of gastric cancer carcinogenesis is thought to be multifactorial, molecular and genome-wide approaches have identified various genetic alterations associated with gastric tumorigenesis and progression [2]. Two types of gastric cancers have been identified on the basis of histological differences, epidemiology, etiology, pathogenesis, and biological behavior. The first type is the diffuse type, which is characterized by infiltrating cells, poor differentiation, and noncohesive cancer cells with vast fibrous stroma, and the second type is the intestinal type, which mostly features cohesive and glandular-like cells [3]. Compared with the intestinal type, the diffuse type is more prevalent among younger individuals, and its metastasis is often identified in the peritoneum or lymph nodes, thereby making prognosis extremely difficult. Currently, diffuse gastric cancer is treated with chemotherapy and targeted therapy by using molecular approaches.

G protein-coupled receptors (GPCRs), which form the largest family of cell surface receptors, have been shown to modulate most physiological functions in the body. However, their roles in cancer development are not clearly understood [4,5]. Recently, P2RY1, a member of GPCRs, has emerged as a cancer target because of its critical role in tumor growth and metastasis. P2RY1 activation via the endogenous agonist ADP is shown to alter multiple physiological functions [6,7,8,9,10]. Interestingly, the P2RY1 receptor is shown to regulate cell growth and death in the following cancer cell lines: SAS oral squamous cells [11], 1321N1 astrocytoma cells [12,13], A375 melanoma cells [14,15], and PC3 prostate cancer cells [16]. Blocking the P2RY1 receptor by using the antagonist MRS2179 reverses cell proliferation, suggesting that the P2RY1 receptor may have an antiproliferative effect [17,18]. Additionally, other P2RY receptor subtypes, such as the Gq-coupled P2RY12 and P2RY14 receptors, have been reported [6,19,20] to regulate cell death and growth [13,21,22,23,24,25]. Studies showed that the levels of P2RY1 mRNA in aggressive gastric cancer tissues are low compared with those in noncancerous gastric tissues. This result indicates that the lack of P2RY1 may contribute to the development of aggressive gastric cancer growth. However, studies on the role of P2RY1 receptor signaling in gastric cancer are lacking. Although most cancers have been linked to specific DNA methylation, epigenetic factors have not been associated with diffuse gastric cancer.

In this study, we have used DNA methylation chip technology and previously published data to analyze the role of the P2RY1 receptor in gastric cancer. Our results show that gastric cancer tissues have high levels of DNA methylation and low levels of P2RY1 receptor protein expression compared with noncancerous gastric tissues. Then, we have used gastric cancer cells to analyze the involvement of P2RY1 receptor in cell death and growth. The subsequent activation of P2RY1 receptor by using a selective P2RY1 receptor agonist, the ADP analog MRS2365 [26], reveals that the receptor induces apoptosis and inhibits cell proliferation. These results indicate that the P2RY1 receptor may be a potential target for the treatment of gastric cancer.

## 2 Materials and methods

### 2.1 Cancer tissues

Tissue samples for the study were obtained from six patients with gastric cancer (2 intestinal- and 4 diffuse-type cancers with matched normal tissue) at the Department of Surgical Oncology of the Zhongshan Hospital in China. Two samples, i.e., gastric tumor samples and noncancerous samples from the adjacent areas, were obtained from each patient. After collection, samples were flash-frozen in liquid nitrogen and stored at −80°C until further use. The QIAamp DNA Mini Kit (QIAGEN) was used to extract DNA from individual samples on the basis of the manufacturer’s protocol.


**Informed consent:** Informed consent has been obtained from all individuals included in this study.
**Ethical approval:** The research related to human use has been complied with all the relevant national regulations, institutional policies, and in accordance with the tenets of the Helsinki Declaration, and has been approved by the Human Research Ethics Committee of Zhongshan Affiliated Hospital of Xiamen University.

### 2.2 Methylation chip experiment

Microarray hybridization and scanning data were conducted in accordance with standard protocols. The whole genome from each tissue received bisulfite treatment and was amplified, fragmented with restriction enzymes, and hybridized to the Illumina Infinium Human Methylation 450 BeadChip kit, which could analyze >450,000 methylation sites that were reported to cover 99% of the genes in RefSeq. These methylated regions were also reported to be distributed along the entire gene, including the transcription start site (TSS), 5′ untranslated region (UTR), 3′ UTR, and exons. In this study, we filtered the TSS and first exon, which were known to mostly regulate a gene. Following hybridization, allele-specific single-base extension and staining were performed. Then, the BeadChips were imaged on the Illumina BeadArray Reader platform. The Illumina’s BeadScan software was used to extract image intensities. Methylated and unmethylated regions in the genome were recognized by the differences in fluorescence intensities, which were marked as data points after subtracting the background fluorescence. The intensities of methylated and unmethylated signals were normalized using the Illumina Genome Studio program. The average methylation (*β*) value was calculated from 30 replicates of methylation data from methylated (Cy5) and unmethylated (Cy3) alleles to array data points. In these analyses, we excluded methylation data from the X chromosome. The filtered methylation sites were then mapped to their potential corresponding gene defined in the UCSC Genome Browser HG19 RefSeq database.

### 2.3 Cell culture

Noncancerous human gastric (GES1) and gastric cancer (BGC823, MKN45, SGC7901, HGC27, AGS1, and MGC803) cells were cultured in RPMI 1640 or DMEM/F-12 medium containing the following antibiotics and supplements: 10% FBS, 100 U/mL penicillin, 100 mg/mL streptomycin, and 3 mM/L glutamine. Cells were maintained at 37°C with a constant supply of 5% CO_2_ in a humidified incubator.

### 2.4 Western blotting

The following protocol was used to extract total proteins from tissues. Tissues collected from patients were lysed for 20 min in 1 mL cell lysis buffer on ice after thoroughly washing with PBS. Then, lysates were centrifuged at 14,000×*g* for 30 min to collect the supernatant. Total proteins from cell cultures were extracted using the RIPA protein extraction reagent (Solabio, Beijing, China) containing a protease inhibitor cocktail (Roche, Basel, Switzerland) in accordance with the manufacturer’s protocol. Then, the total protein concentrations in tissue supernatants and cell lysates were measured using the BCA method. About 30 μg total proteins from each tissue sample and 50 μg total proteins from each cell culture were resolved using 10% SDS-PAGE, transferred into polyvinylidene fluoride (PVDF) membranes (Millipore), and processed for Western blotting. Briefly, anti-P2RY1 (1:1,000) was used as primary antibody (Sungon, China), and the goat anti-rabbit IgG (1:5,000) was used as secondary antibody (Santa Cruz Biotechnology, CA). GAPDH was used as control to verify the loading of equal quantity of proteins. Protein bands were visualized by the ECL-based chemiluminescence signals, which were recorded using the ImageQuant LAS 4000 mini instrument. SGC7901 cells were first cultured for 4 h in serum-free medium and incubated in serum-supplemented medium to which various pharmacological reagents were previously added to detect phosphorylation. Cells were cultured in these conditions at 37°C and at various time points. Then, the medium was replaced with 100 ml cold RIPA buffer containing proteinase inhibitors to terminate the reactions. SGC7901 cells (2 × 10^5^ cells per well) were grown to 70% confluence in 6-well plates to detect caspase-3 activity. Then, apoptotic inducers (Beyotime, China) were added to the cells 60 min before treatment with the P2RY1 receptor agonist, MRS2365, for 8 h. Control groups did not receive apoptotic inducers.

### 2.5 Cell viability assay

SGC7901 cells at a density of 1 × 10^4^ cells were cultured overnight in 100 μL medium containing 10% serum per well in a 96-well plate at 37°C to investigate the cell viability. Then, MRS2365 (1 mM) was added to the cells and placed in an incubator maintained at 5% CO_2_ and 37°C for 24, 48, or 72 h. The control group was not treated with MRS2500. The MTT Toxicology Assay Kit (Sigma-Aldrich, St. Louis, MO) was used to assess cell viability in accordance with the manufacturer’s instructions. Cell viability was quantified by measuring the absorbance at 560 nm on MKV reader (Thermofisher, CA). Cell viability was estimated as a percentage of live cells in the treated groups vs the control.

### 2.6 Cell cycle distribution

About 1 × 10^5^ SGC7901 cells per well in a 6-well plate were treated with MRS2365 for 36 h to determine cell cycle arrest by MRS2365. Then, the medium was removed, and cells were fixed overnight in 70% ethanol at 4°C. Cell cycle distribution was analyzed after staining with propidium iodide (PI) (100 μg/mL, Sigma-Aldrich) by using the Guava easyCite flow cytometer (Millipore).

### 2.7 Cell migration assays

The transwell system with an insert (8 μm pore size; Millipore, Bedford, MA, USA) was used to perform migration assays. In the upper chamber, 5 × 10^4^ cells were plated in serum-free medium and added in the lower chamber medium supplemented with 10% FBS. Then, 1 mM MRS2365 was added to the upper chamber, and cells in the lower chamber that migrated through the insert were detected by staining with 0.5% crystal violet after 24 h. The cells remaining on the upper part of the insert were removed with cotton wool and analyzed. All cells were then visualized through the ZEISS microscope (New York, USA) to obtain pictures, and cells in six random fields per well were counted and used to estimate cell migration.

### 2.8 Apoptosis assay

Apoptosis was measured using the FITC Annexin V Apoptosis Detection kit (BD Pharmingen™, USA) in accordance with the manufacturer’s instructions. SGC7901 cells were cultured in the presence or absence of apoptotic inducers from the Apoptosis Inducer Kit (Beyotime, China), harvested, mixed with 500 μL Annexin V binding buffer, and incubated with 5 μL of each Annexin V-FITC and PI for 15 min in the dark. Positive staining was analyzed using the Guava easyCite flow cytometry (Millipore, USA).

### 2.9 Statistical analysis

All data were presented as mean value ± standard deviation (SD). Statistical analysis was performed using the R 2.3.0. Statistical differences (*p < 0.05) between treated and control groups were analyzed using a two-tailed *t*-test.

## 3 Results

### 3.1 Hypermethylation of P2RY1 DNA in the promotor region that lowers protein expression in gastric cancer tissues

**Figure 1:**
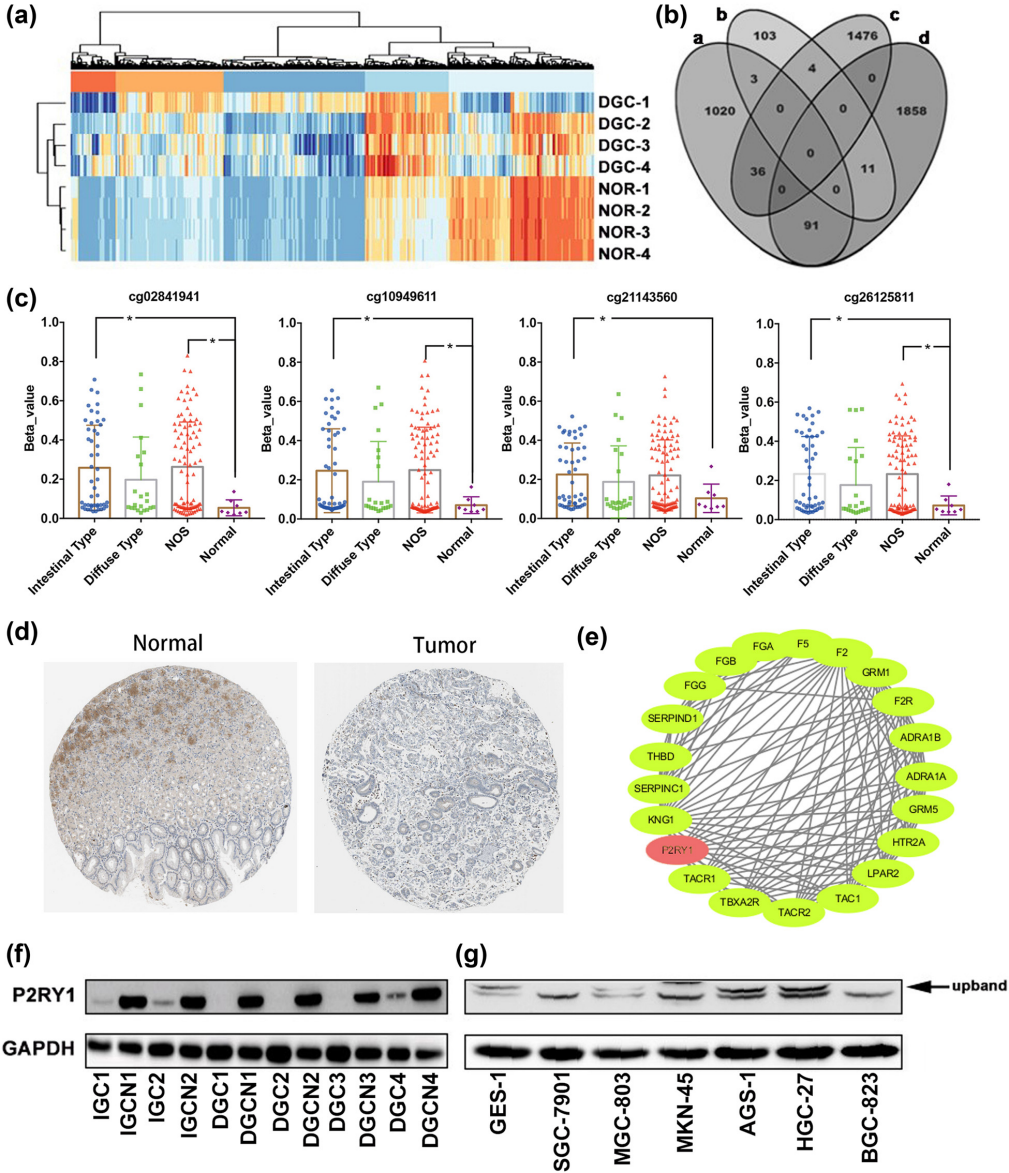
Chip data and Western blot analyses of P2RY1 receptor expression in human intestinal and diffuse gastric cancers and endogenous P2RY1 expression in gastric cancer cell lines. (a) Hierarchical clustering of differentially methylated sites in gastric cancer tissue compared with those in normal tissue. (b) Venn diagram depicting the overlap between differentially expressed genes in chip methylation and mRNA expression data (published). The cutoff value for a significant methylation site is the |Δ*β*| > 0.2. “a” represents genes with low mRNA expression in reference, “b” represents genes with high mRNA expression, “c” indicates genes that are hypomethylated in the chip experiment in this study, and “d” indicates genes that are hypermethylated in the chip experiment in this study. (c) Distribution of *β* shown in accordance with four high methylation sites. Data about beta value are shown as mean value ± SD. *p < 0.05. The number of normal, intestinal, diffuse, and NOS types are 8, 48, 22, and 97, respectively. (d) Protein levels of P2RY1 in normal stomach (staining: low; intensity: moderate) and stomach tumor (staining: not detected, intensity: negative) tissues. (e) PPI networks of P2RY1 and its associated proteins. (f) Expression of P2RY1 protein in the intestinal/diffuse gastric cancer tissues and the corresponding noncancerous tissues by using Western blotting. (g) P2RY1 protein expression in gastric cell lines by using Western blotting.

We performed the DNA methylation chip experiment to establish the methylation spectrum of human diffuse gastric cancer (Figure 1a). Then, our data were validated/extended and compared with previously published results [27] by constructing a Venn diagram of methylated genes and mRNA that were significantly different between gastric cancer and noncancerous tissues (Figure 1b). Among the genes analyzed, P2RY1 had four high methylated sites (Table 1) within its promoter region, whereas its mRNA level was low in gastric cancer tissues. After comparing with TCGA/STAD database, the four sites in the gastric cancer samples also showed high methylation level status compared with those in normal gastric samples in Figure 1c. In this study, two and four samples were identified histopathologically as intestinal and diffuse gastric cancers, respectively. Immunohistochemical staining data obtained from the HPA database demonstrated the downregulated expression of proteins encoded by P2RY1 in stomach cancer tissue (Figure 1d). P2RY1 was analyzed by the STRING database and Cytoscape software. As shown in Figure 1e, PPI was composed of P2RY1 and its associated proteins. The expression of P2RY1 differed markedly among the two sample types with diffuse gastric cancer tissues showing significantly lower level protein expression of P2RY1 compared with intestinal gastric cancer tissues (Figure 1f). Thus, the low expression of P2RY1 appeared to be a critical factor in establishing diffuse gastric cancer. Moreover, we analyzed the expression levels of P2RY1 in various gastric cancer cell lines by Western blotting to determine how P2RY1 affected the development of gastric cancer. Among all the lines tested, SGC7901 had high P2RY1 protein level, and BGC823 had low P2RY1 protein level (Figure 1g). On the basis of these results, SGC7901 cells were chosen for further experiments for P2RY1 function.

**Table 1:** Methylation loci sites within P2RY1’s TSS region

TargetID	Δ*β* value	Gene location	Chromosome
cg02841941	0.28431	TSS200	chr3
cg10949611	0.28524	TSS200	chr3
cg21143560	0.20043	TSS200	chr3
cg26125811	0.26865	TSS200	chr3

### 3.2 P2RY1 receptor-mediated activation of ERK1/2 signal pathway

**Figure 2:**
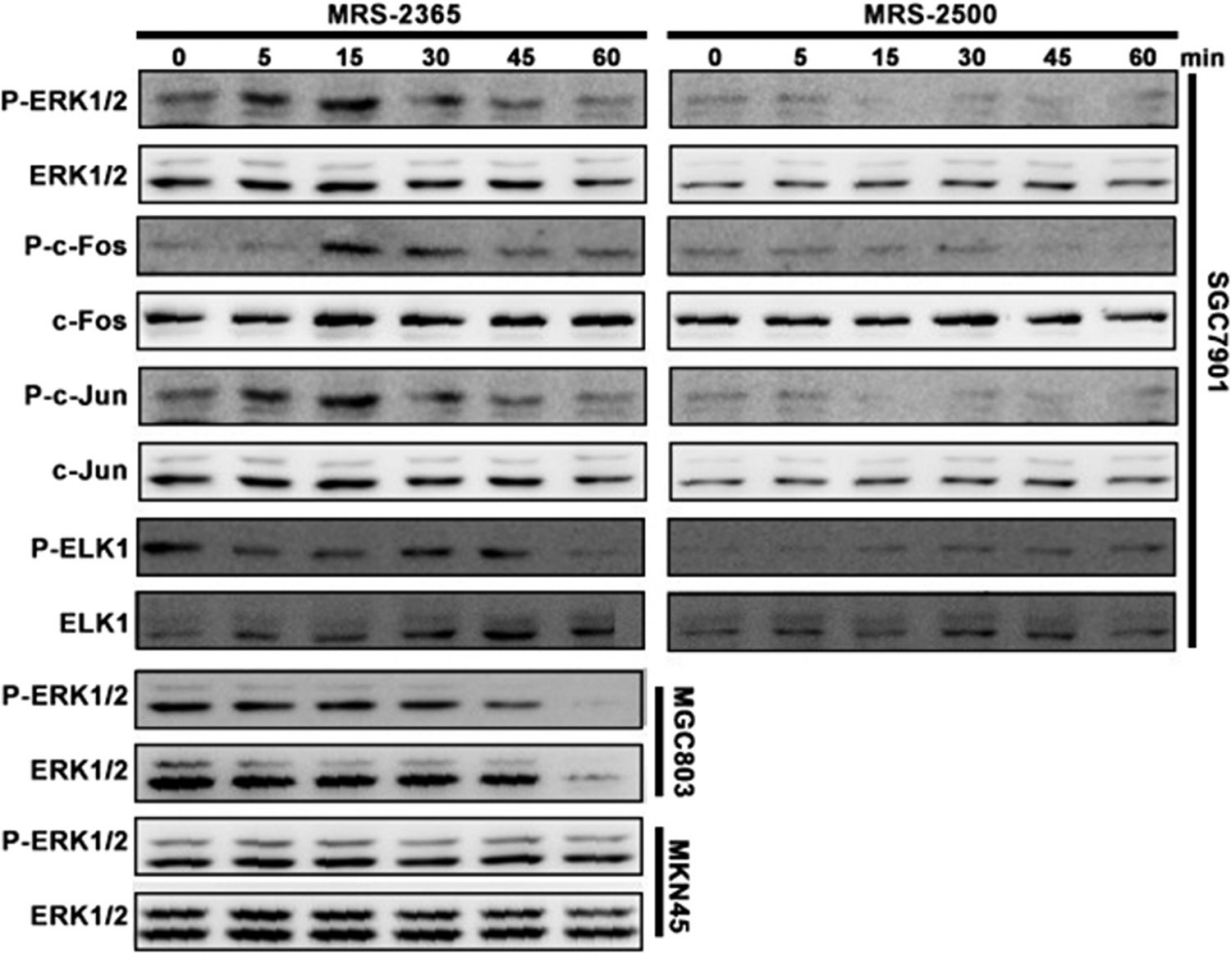
Promotion of ELK1/c-Fos/c-Jun activation through the MRS2365-induced activation of ERK1/2 in SGC7901 cells. SGC7901 cells were treated with 10 ng/mL MRS2365 and MRS2500 at 37°C for 1 h.

The treatment of SGC7901 cells with the P2RY1 agonist, MRS2365 (1 mM), increased ERK1/2 phosphorylation and ELK1/c-Fos/c-Jun phosphorylation to the maximum in the first 5 min followed by a decrease in the basal level within 15 min (Figure 2). By contrast, the treatment of MGC803 and MKN45 cell lines (high levels of P2RY1) with MRS2365 did not result in an increase in ERK1/2 phosphorylation. These results indicated that the P2RY1 agonist, MRS2365, activated the ERK1/2 signal pathway in gastric cell lines with P2RY1 protein.

### 3.3 Inhibition of the proliferation and metastasis of gastric cancer cell through the activation of the P2RY1 receptor

**Figure 3:**
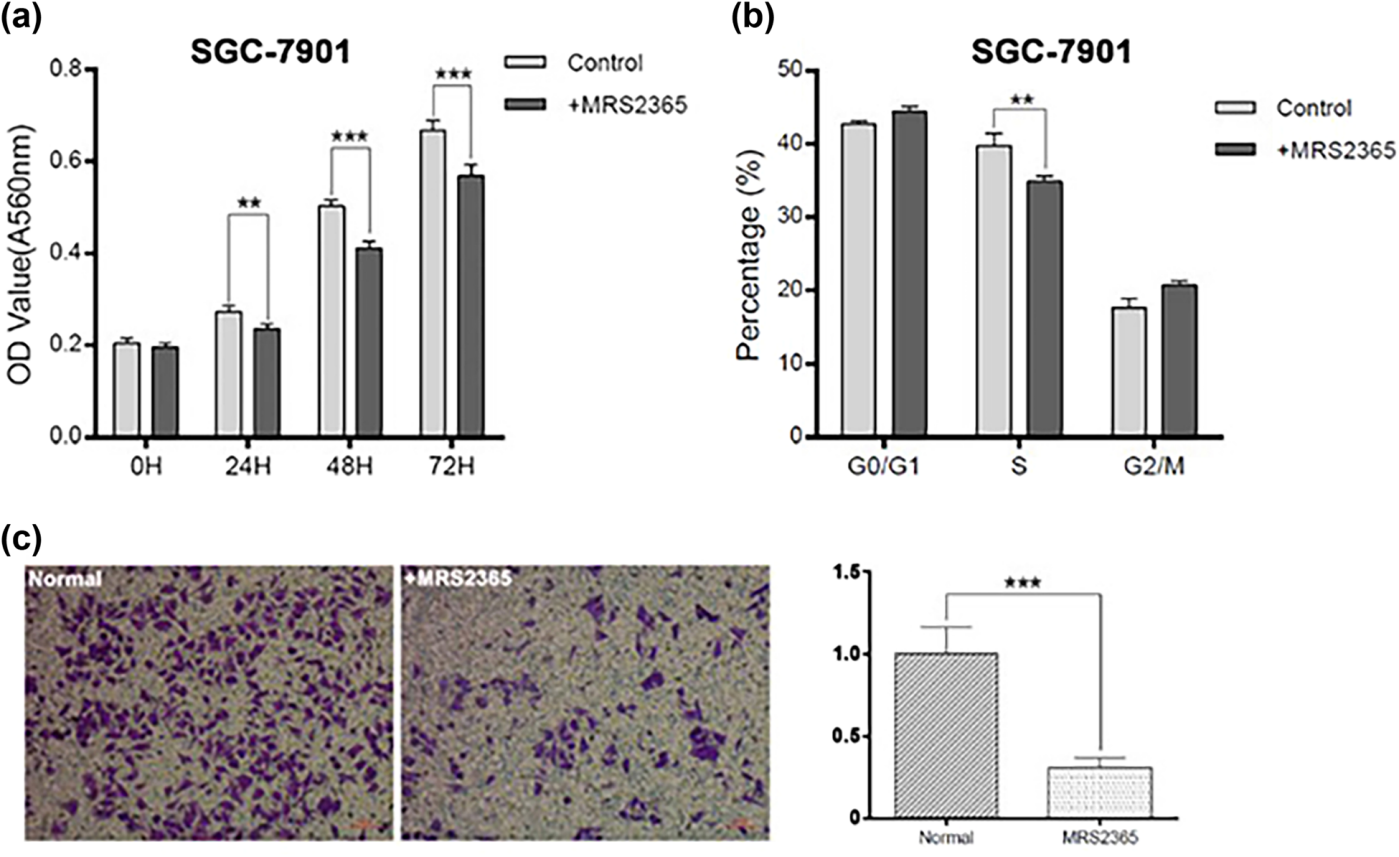
Effects of MRS2365 on the proliferation and migration of SGC7901 gastric cancer cells. (a) MTT assay. SGC7901 cells were cultured with MRS2365 (1 mM) in 96-well plates for 3 days. Controls did not receive MRS2365. Then, MTT was added, and MTT activity was measured after 4 h at 37°C. Control cells did not receive MRS2365. All experiments had three replicates. Data are presented as mean value ± SD. (b) Effect of the P2RY1 agonist on the cell cycle. SGC7901 cells were grown overnight to ∼30% confluence in complete RPMI 1640 medium in 6-well plates and treated with MRS2365 (100 nM) for 24 or 72 h. Cell cycle analysis was carried out with PI staining. MRS2365 caused G1 cell cycle arrest. Data represent the results of two independent experiments. (c) Flow cytometry results from three independent experiments. Data were analyzed by Student’s *t*-test. Data represent mean value ± SD. **p < 0.01; *p < 0.05.

To investigate whether the activation of the P2RY1 receptor resulted in an antiproliferative effect, we used the MTT assay to determine cell proliferation. Results showed that treatment of SGC7901 cells with MRS2365 for 24, 48, or 72 h significantly lowered cell numbers than in the control group (Figure 3a; *p* < 0.05, paired test), indicating an antiproliferative effect. To determine the mechanism by which MRS2365 inhibited the growth of SGC7901 cells, we determined the stages of cell cycle arrest in treated cells by using flow cytometry. After 24 h of treatment, we found that 34.9% (±0.81) of the cells were in the S phase compared with 39.7% (±1.75) in the control group (*p* < 0.05, Figure 3b). This result indicated that MRS2365 might inhibit cell growth by arresting the cell cycle in the G2 phase. We then used the transwell system to investigate the effect of P2RY1 on gastric cancer cell migration. We found that the activation of P2RY1 decreased SGC7901 cell migration (Figure 3c). These results suggested that the activation of the P2RY1 receptor could partly inhibit cell proliferation/metastasis.

### 3.4 Effect of P2RY1 receptor activation on apoptosis

**Figure 4:**
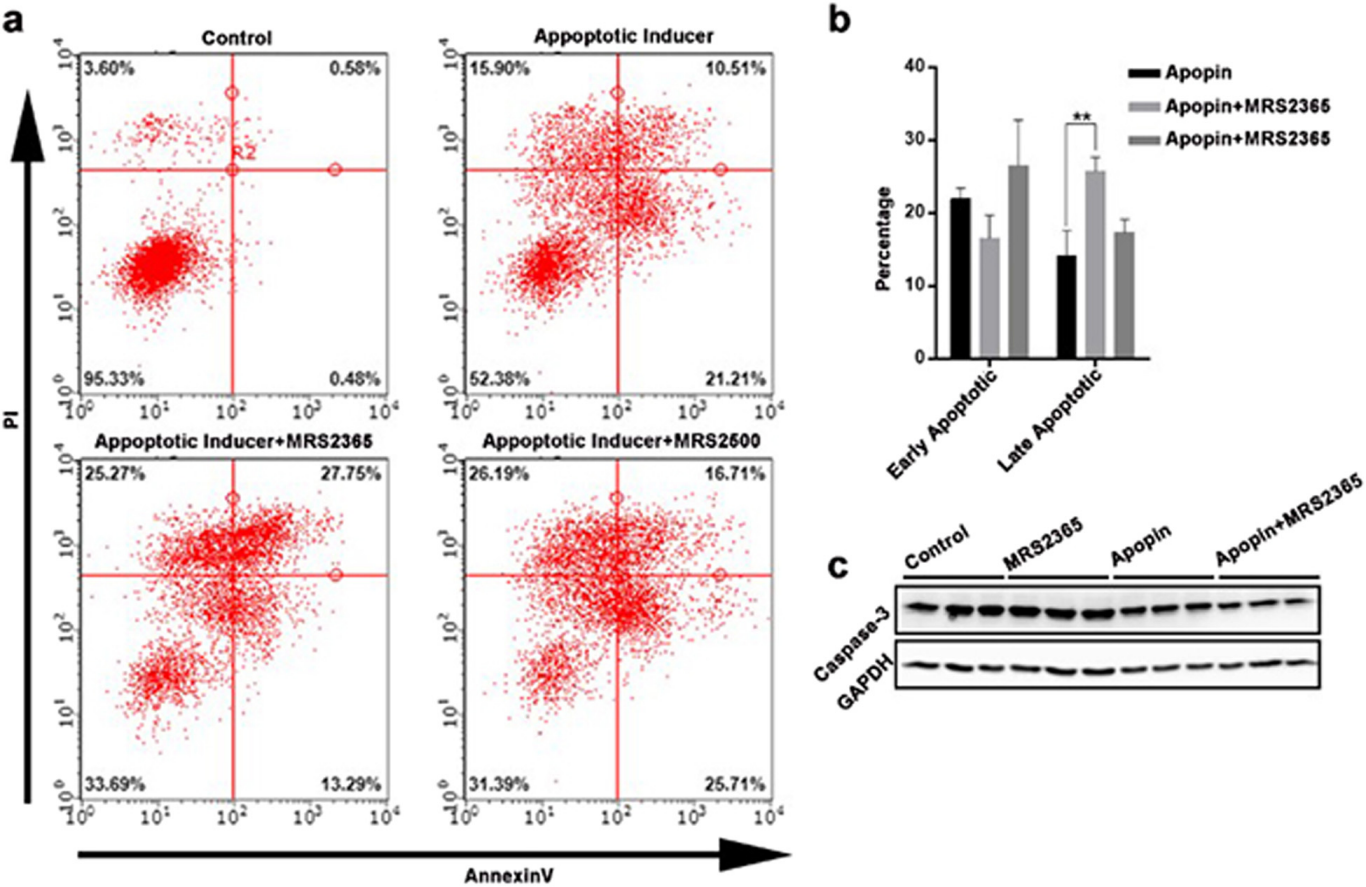
Induced apoptosis in SGC7901 cells by P2RY1 receptor activation. (a) Cells were stained with annexin V and propidium iodide to determine apoptosis by flow cytometry. Viable cells stained negative for annexin V and PI (lower left quadrant), cells in early apoptotic stage stained positive for annexin V but negative for PI (lower right quadrant), and necrotic cells or those in late apoptotic stage stained positive for annexin V and PI (upper right quadrants). (b) Mean value ± SD of three independent experiments. **p < 0.01, control vs MRS2365-treated group. (c) Western blot analysis of caspase-3 expression. Proteins were extracted from cells treated with MRS2365 (1 mM) in the presence of apoptotic inducer for 8 h. Proteins were separated on SDS-PAGE, blotted onto PVDF membranes, and probed with anti-caspase-3 antibodies. GAPDH served as the reference for the loading of equal quantity of proteins. Asterisks represent protein expression that is significantly different compared with the apoptotic inducer + MRS2365 group.

P2RY1 receptor activation is known to induce apoptosis in PC3 prostate cancer cells [28]. Therefore, we examined the effect of P2RY1 activation via the MRS2365 agonist on the apoptosis of SGC7901 cells by measuring the caspase-3 activity (Figure 4a and b) as described previously [29]. As shown in Figure 4c, MRS2365 (1 mM) promoted caspase-3 activity in SGC7901 cells cultured with apoptotic inducers. By contrast, MRS2365 did not increase caspase-3 activity in the absence of apoptotic inducers.

## 4 Discussion

Over the past 50 years, gastric cancer incidence and related mortality have decreased significantly worldwide. However, in the 2020 Global Cancer Data Report, patients with cancer who died of gastric cancer ranked fourth. Also, in Asia, gastric cancers remain prevalent and result in loss of life in large numbers. This finding is largely due to poor prognosis, which results in metastasis and subsequent death [1]. In this study, we have found that the P2RY1 receptor may play a critical role in causing gastric cancer. Our results have shown that four higher methylated sites of P2RY1’s promoter region can be used as markers for the prognosis of gastric cancers.

**Figure 5:**
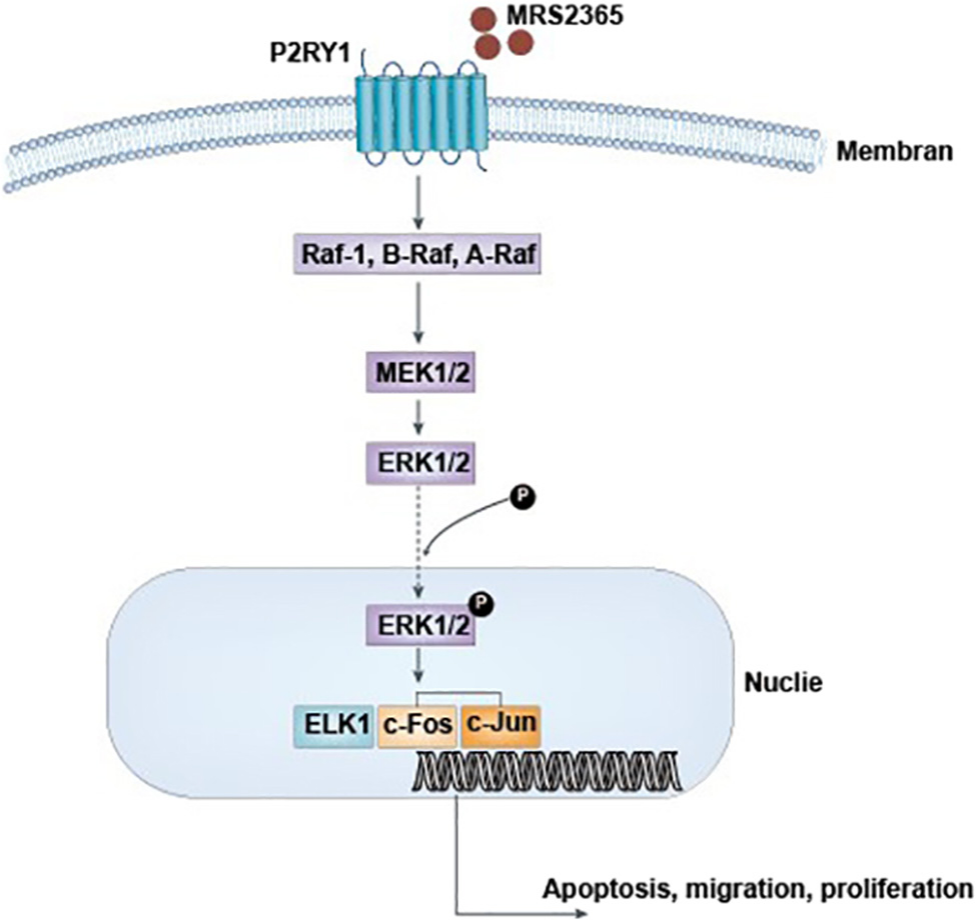
Effect of the MRS2365 on the Ras–Raf–MEK–ERK signaling pathway. In human tumors, the MAPK pathway is activated by P2RY1 agonist (MRS2365) binding to the P2RY1 receptor. The sequential activation of Ras–Raf–MEK–ERK is known to phosphorylate nuclear targets (C-Fos, ELK1, and c-Jun) that cause apoptosis, migration, and proliferation of cells. Each tier in the pathway consists of 3 Rafs (1, A, and B), 2 MEKs (1 and 2), and 2 ERKs (1 and 2). The phosphorylation of ERK eventually controls the transcription of genes that promote cell cycle progression and tumor survival. This result is critical because a number of genes have been reported as targets of ERK.

In the diverse MAPK signaling pathway, the expression of early genes, such as c-Fos, c-Jun, and Elk1, is regulated by the phosphorylation of transcription factors [30,31] c-Fos and c-Jun phosphorylation and increases astrocyte proliferation and GFAP expression. Besides, *in vitro* and *in vivo* studies showed that ATP can upregulate the expression of the c-Fos protein and the formation of AP-1 transcriptional complexes [32,33]. In the present study, we found that P2RY1 receptor activation in SGC7901 can induce apoptosis. This result is consistent with previous reports after the activation of the P2RY1 receptor [12,13,14,15,16]. Previous studies showed that the P2RY1 receptor-mediated apoptosis in astrocytoma cells (1321N1) and prostate cancer cells (PC3) is correlated with ERK1/2 activation. Our study further examined the potential involvement of MAPK signaling in gastric cancer cells. We showed that the selective activation of P2RY1 via its agonist, MRS2365, can induce ERK1/2 phosphorylation and subsequent ELK1/c-Fos/c-Jun phosphorylation, suggesting the crucial role of ERK1/2 signaling in gastric cancer cells (Figure 5). The Ras–Raf–MEK–ERK pathway activation is known to be critical for the proliferation of many human tumors [34,35,36]. Thus, this pathway may serve as an important molecular target for anticancer therapy. Specifically, the P2RY1 receptor may serve as a novel anticancer target.

Besides inducing apoptosis, the inhibition of cell proliferation is a function of P2RY1 receptors. The antiproliferative effect of P2RY1 receptors is first demonstrated in 1321N1 astrocytoma cells that express a recombinant human P2RY1 receptor [37]. We also found that the selective activation of P2RY1 receptors by using the agonist, MRS2365, can inhibit SGC7901 cell proliferation. This finding is consistent with the previous studies [14,15].

In previous studies, the expression of the P2RY1 receptor is low in gastric cancer tissues [38]. In this study, we also showed the beneficial effects of the P2RY1 agonist, which has been shown in other cancer models, such as melanoma, where the P2RY1 receptor is highly expressed [15]. The enhanced *in vivo* stability of the dinucleotide P2RY1 agonists also increases their potential use to treat other diseases [39].

## 5 Conclusion

In summary, we have demonstrated that gastric cancer tissues have highly methylated P2RY1 gene and relatively low expression level of P2RY1 mRNA compared with noncancerous tissues. We also showed that MRS2365, the selective agonist of the P2RY1 receptor, can induce ERK1/2 phosphorylation, which may inhibit cell proliferation/migration and induce apoptosis. These findings suggested that P2RY1 plays an important role in the development of gastric cancer. Finally, our results underscore the potential therapeutic application of P2RY1. The proapoptotic and antiproliferative effects induced by P2RY1 receptor activation indicate that the P2RY1 receptor may be an attractive target for the treatment of gastric cancer.
