# Erratum to “Activation of hypermethylated P2RY1 mitigates gastric cancer by promoting apoptosis and inhibiting proliferation”

**DOI:** 10.1515/biol-2023-0001

**Published:** 2024-07-24

**Authors:** Yinggang Hua, Yanling Liu, Long Li, Guoyan Liu

**Affiliations:** Department of Basic Medicine, Medical College of Xiamen University, Xiamen, Fujian, China; School of Pharmaceutical Sciences, Xiamen University, Xiamen, Fujian, China; Department of Gastrointestinal Surgery, Zhongshan Hospital Xiamen University, Xiamen, China

In the published manuscript, “Activation of hypermethylated P2RY1 mitigates gastric cancer by promoting apoptosis and inhibiting proliferation. Yinggang Hua, Yanling Liu, Long Li and Guoyan Liu, Open Life Sciences. 2023;18(1): 20220078. https://doi.org/10.1515/biol-2022-0078,” the authors have found an unintentional error made during preparation of Figure 2. The statement from the authors reads as follows:

During our recent review of the experiments described in the article, we identified an issue. Specifically, in Figure 2 of the article, there is an error in the Western blot (WB) images of P-c-Jun and c-Jun. This error occurred during the figure preparation process, where the WB images of P-ERK1/2 and ERK1/2 were mistakenly placed in the positions for P-c-Jun and c-Jun, resulting in repeated images. Figure 1 in this letter highlights the erroneous information.

**Figure 1:**
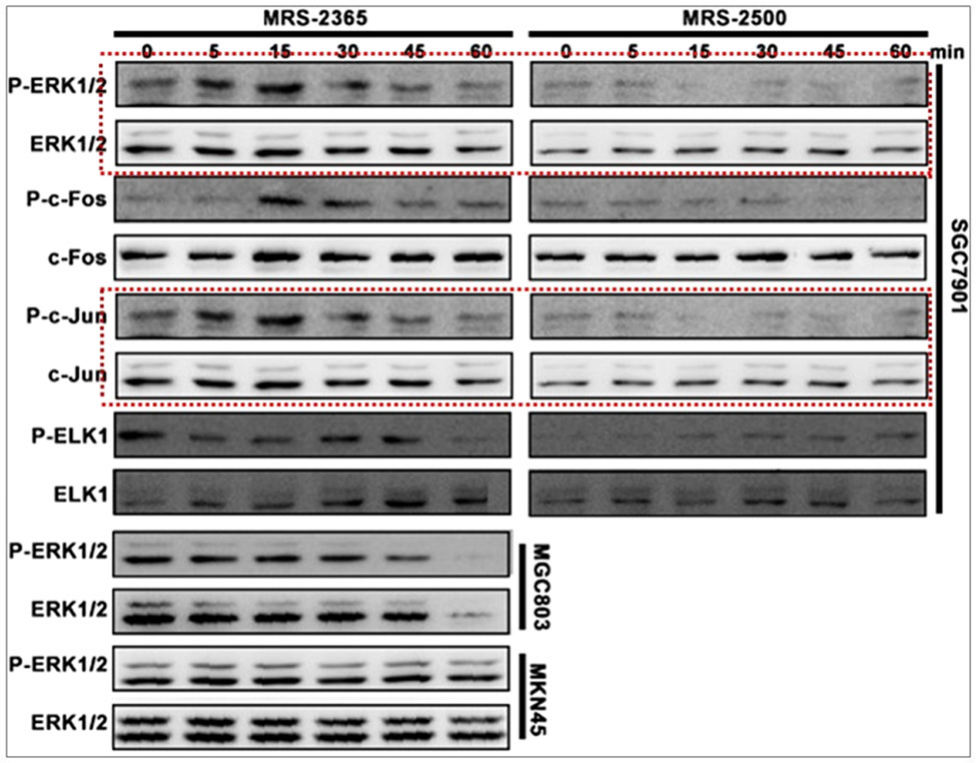
Red box indicates the repeated images of P-c-Jun and c-Jun in Figure 2 of the article.

Figure 2 presents the actual WB results for P-c-Jun and c-Jun (MRS2365 agonist and MRS2500 antagonist).

**Figure 2:**
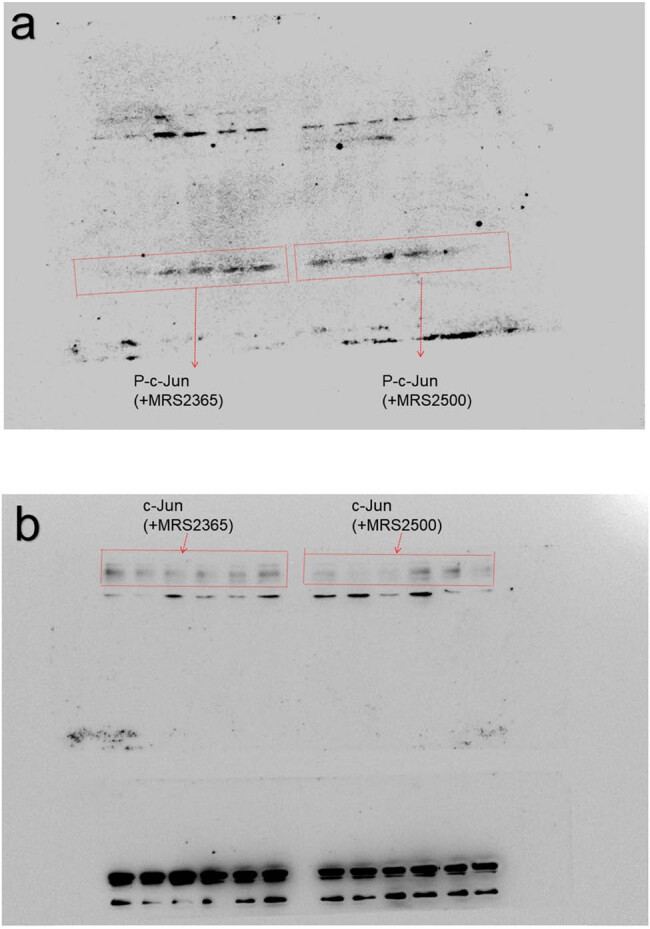
(a and b) The WB results for P-c-Jun and c-Jun (MRS2365 agonist and MRS2500 antagonist).

Our experimental results indicate that the protein level of P-c-Jun increases after stimulation of SGC-7901 cells with MRS2365 (agonist), suggesting the phosphorylation and activation of c-Jun in the signaling pathway. Based on these accurate and reliable experimental results, we have created a new version of Figure 2 from the original manuscript. The new Figure 2 (here as Figure 3) corrects the WB results for P-c-Jun and c-Jun (MRS2365 agonist and MRS2500 antagonist). The corrected image does not affect the core content and conclusions of the original paper.

**Figure 3:**
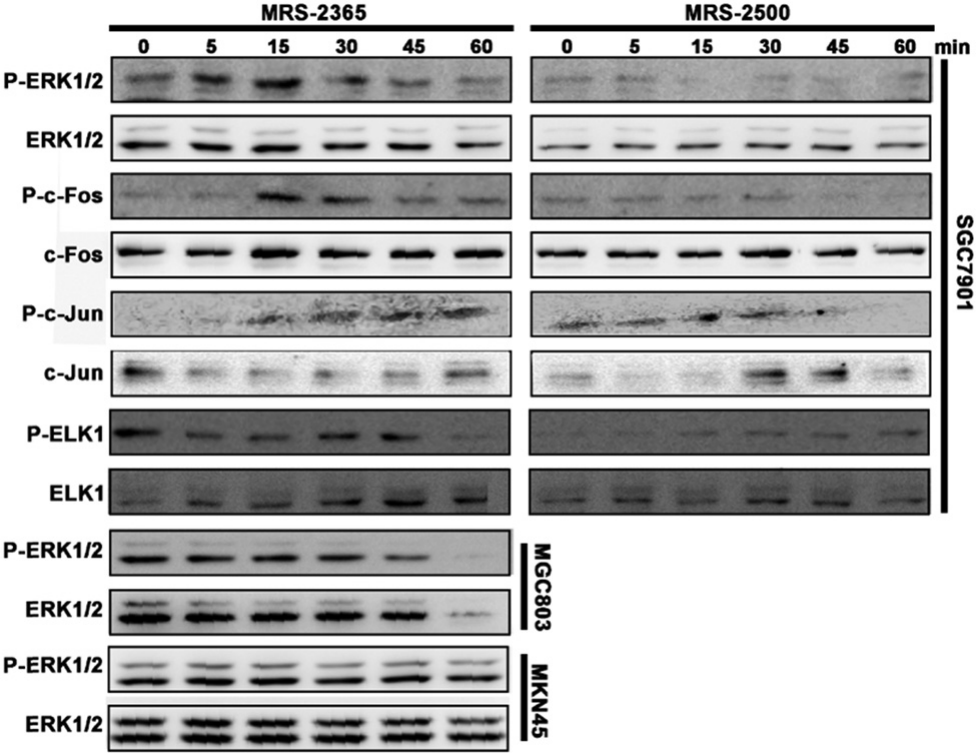
Corrected version of Figure 2 in the article.

The authors admit the error and claim that this is an unintentional error that has nothing to do with academic misconduct and does not influence the conclusion of the publication. The authors apologize to the editor, the staffs of the journal, and the journal readers for the mistake and any inconvenience it caused.

